# The role of community protection institution in disaster management at West Java, Indonesia

**DOI:** 10.4102/jamba.v13i1.943

**Published:** 2021-08-03

**Authors:** Etin Indrayani, Sadu Wasistiono

**Affiliations:** 1 Public Security and Safety Management, Governmental Management, Institute of Governance of Home Affairs, Sumedang, Indonesia; 2 Governmental Science Post Graduate Program, Governmental Management, Institute of Governance of Home Affairs, Sumedang, Indonesia

**Keywords:** community protection, disaster management, Linmas, Protection Unit, self-security

## Abstract

This research aimed to establish a mechanism of evolution of community protection, identify the support of facilities and infrastructure needed in facilitating the implementation of the tasks and functions of Linmas, especially in disaster management and formulate community protection institutions that are appropriate to the needs and capacities of the regions. The research method is carried out by a qualitative approach that is using focus group discussion (FGD) based on experience and perceptions of the benefits and impacts of the community protection unit’s guidance in West Java province. The results show that the community protection institutions are needed in improving the independence of the community in tackling any disaster that is faced by an organization in which at least have the ability and skills in the field: early prevention, peace and orderly of safety fibre, health and psychologist and public and social work. The practical implication of this research is that the local government should empower the community protection unit through the regional work unit or related stakeholders in conducting training and facilitation of training and improving skills so that they can carry out their duties better.

## Introduction

The community protection activities (Linmas) through the establishment of task forces (Satgas) have existed in various regions in Indonesia. The formation of *Satlinmas* itself experienced a relatively long historical journey to the present before the establishment of *Satlinmas* was often also referred to as Civil Defense (*Hansip*). According to Bulo (2014) speaking of *Satlinmas*, we must return to the first regulation on Civil Defense (*Hansip*), namely the Decree of the First Deputy Minister for Defense/Security Number MI/A/72/62 dated 19 April 1962. Next, through a Presidential Decree Number 56 of 1972, guidance, which was previously under the Departement of Defense/Security at the time, was handed over to the Department of the Interior.

In line with the development of the life of the state administration and governance nationally, the position and role of *Hansip* have changed to become Linmas by Circular of the Minister of Home Affairs Number 340/2921/SJ dated December 20, 2002, with the main tasks and functions of assistance in disaster management, social activities community, post-conflict local election and assisting other duties stipulated in the legislation. As an illustration of the main task of *Hansip* when under the auspices of the Department of Defense Security covers security and defense whilst in Linmas interpreted as a function in protecting the community (Gunawan 2015). A community protection unit (*Satlinmas*) is a community member prepared and equipped with knowledge and skills to carry out disaster management activities to reduce and minimise the impact of disasters and to maintain security, peace and public orderly and social events. Members of the community protection unit (*Satlinmas*) are eligible citizens and voluntarily participate in community protection activities. The implementation of community protection is the organising and empowerment of community protection. However, the existing Linmas are considered not useful because of management constraints, facilities, funds and coaching that have not been directed.

In the case of the forest fire, Saptawan et al. (2019) said that the number of Linmas had contributed significantly to the odds of forest fires in Sumatra Island. The number of ‘hansip’ officers as predictors of forest fires that have not received the attention of forestry researchers in Indonesia. This finding shows that the efforts of government and private corporations to strengthen community institutions in preventing and combat forest fires are in the right location and/or village. Although the existing literature emphasises the vital role local governments play in introducing, managing and implementing disaster risk reduction (DRR) initiatives, local-level institutions are not yet fully empowered, generally in developing countries. Currently, attention is directed to local governments in managing disasters as they play an active role in disaster-related activities in collaboration with communities (Kusumasari, Alam & Siddiqui 2010).

Disaster management institutions, especially at the local level, can play a significant role in effective DRR with support from district authorities and the community. Furthermore, studies that have been carried out by (Madan & Routray 2015) pointed that although there have been measures undertaken by the institutions for preparedness; however, the institution still faces hurdles in implementation at the institution level. Direct linkages need to be developed amongst all institutions managing disasters to achieve effective DRR, especially local-level institutions and the community heads as the focal point to ensure that institutions work in a complementary manner for enhancing the preparedness to respond to emergencies. It becomes imperative for the institutions, especially at the local level, to be involved in preparedness activities through a participatory approach with the community.

Effective and decentralised policies for DRR can significantly reduce the loss of life and assets caused by disaster (Scott & Tarazona 2011; Williams 2011). The existing research on the impacts of decentralisation of disaster management institutions envisions the procedure positively regarding public service delivery (Pearce 2003) because nations with decentralised government processes experience fewer disaster-related death (Toya & Skidmore 2010). Such governments prepare for and respond to catastrophe more effectively relative to more centralised systems. Also, decentralisation reduces disaster-induced deaths by enhancing human capital (Kahn 2012).

Disaster risk reduction is not just a process of identification, assessment and management of disaster risk. It is also a process of understanding people’s perception about their risk and vulnerabilities, their interaction with each other and indigenous coping strategies, power structures along with laying out the methods of practical cooperation. Prevention and mitigation need to be understood as social phenomena. An effective disaster response entails more than resource management, evacuation, shelter and health interventions; it also rests on an understanding of human behaviour, stresses, strains and vulnerability. Similarly, post-disaster recovery is not merely a mega-project involving construction and rehabilitation. Community-based disaster management activities serve as increasingly important elements of vulnerability reduction and disaster management strategies at the local levels (Allen 2006).

Ainuddin et al. (2013) described the institutional mechanism of Pakistan. The relationship between the different levels is essentially starting from national to union council level. The disasters are managed and handled only at the provincial level (top-down approach), but catastrophe must be treated at all levels, including district, union council and community levels. The implementation of disaster management institutions at the local level in Baluchistan so that communities at the district and sub-district levels engage in the planning and execution of disaster management and risk reduction strategies. Those communities always respond first to any disaster’s impacts; therefore, their role becomes imperative that they are engaged in disaster-related activities at the local level. That is, they are involved in decision making, preparedness and mitigation activities, design, implementation and evaluation of the risk management activities at the community and union council level. Furthermore, the effective implementation of mitigation strategies requires the incorporation of the local decision-making in disaster management processes.

The Hyogo Framework recognised the importance of awareness and preparedness in enabling communities to respond and recover from disaster, which has underpinned most DRR initiatives over the last decade (IFRC 2012). Societies reserve the right to make every effort to reduce the risk and impact of disasters. Community groups in developing countries have applied many successful examples. The social communities had to play in coping with disasters. Moreno’s finding establishes that they have the power to activate internal resilience capacities to deal with and recover from naturally affected cities by accident. The study highlights that communities are not merely passive victims of disasters. By using the role of social networks, organisation, cooperation, trust, local knowledge and participation are active agents (Moreno 2018).

According to Pal, Ghosh and Ghosh (2017), there are several critical issues and challenges faced by institutions engaged in disaster management to reduce the potential impact of future disasters. Results reflect that some factors such as awareness and perception, financial resources, technical resources, policy, institutional arrangements, leadership and human resources help in institutional preparedness and timely response to disasters. Results reflect that some factors such as awareness and perception, financial funds, technical support, policy, institutional arrangements, leadership and human resources prevent useful and timely institutional preparedness and response to disasters. The local institutions and the community being at the forefront, following a participatory approach for readiness. Disaster management in India extends from national to a local level involving multiple stakeholders. In this structure, each previous level guides the activities and decision making at the next level hierarchically. The relationship amongst various institutional stakeholders at different levels is essential because they are interlinked with each other regarding their roles and functions (Pal et al. 2017).

On the other hand, in addition to the existence of institutions, the relevant policy also determines the success of disaster management involving the community. Siriporananon and Visuthismajarn (2018) had identified the key to the success of disaster management in terms of public policy based on a case study of the Hat Yai Asian Cities Climate Change Resilience Network (ACCCRN) to propose guidelines for sustainable disaster management in the area. The result showed that the model of disaster management in Hat Yai, Songkhla Province, involved three critical success factors of disaster management policy: (1) there is a balance between self-interest and the public interest, (2) private participation is required, (3) obstacles to policy implementation and its practical implementation are addressed. The benefits of this research are enormous in terms of successfully implementing a disaster management policy and this policy can be applied to other contexts in Thailand as well.

Comprehensively, Kusumasari et al. (2010) explained that the capability in managing disaster is reflected as a function of institutional resources, human resources, policy for effective implementation, financial and technical resources and leadership. Also, the operation of capabilities is transformed into the critical success factor of disaster management. Essential elements of success are competitive factors that affect the local government’s ability to manage the disaster, as illustrated in the following model.

**FIGURE 1:**
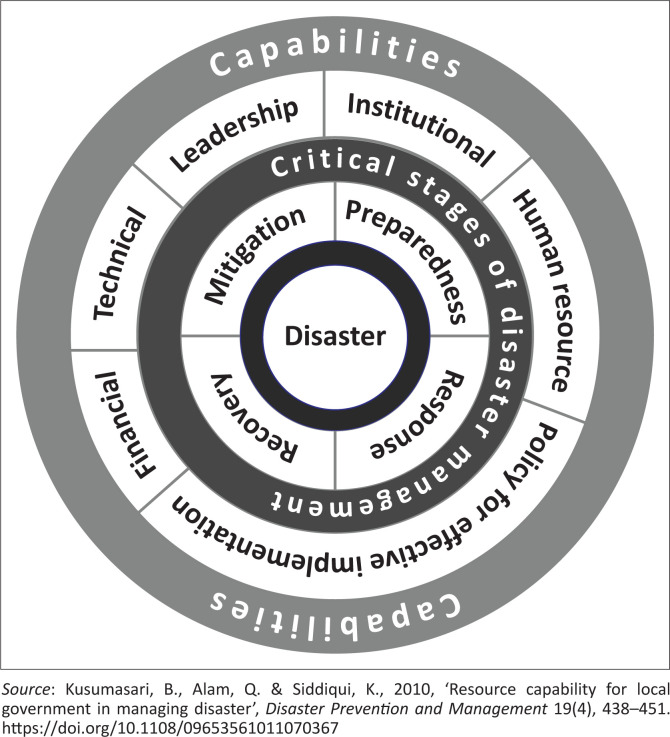
Local government capability in managing natural hazards.

The competitive factors of human resource-related capability are apparent when local government has sufficient personnel, proper tasks, delegation and division of labour within the organisation to manage the disaster (Figure 1). The critical success factors contributing to policy for useful implementation-related capability are the availability of appropriate policies, rules and regulations for making decisions, mobilising resources and engaging relevant public or private organisations. Furthermore, local capability requirements and critical factors of disaster management are briefly illustrated in Table 1.

**TABLE 1:** Local capability requirements and critical factors of disaster management.

Number	Local government capability	Key functional success factors
1.	Institutional	Having a clear structure, rule, responsibilities and relationship between all levels of government
2.	Human resources	Having sufficient personnel, proper task delegation and division of labour
3.	Policy for effective implementation	Availability of appropriate policies rules and regulations for making a decision, mobilising resources and engaging relevant public or private organisations
4.	Financial	Having sufficient financial resources to support activities in all stages of disaster management
5.	Technical	Having an effective logistic management system, sufficient technology information system and communication network between organisations, communities and media representatives
6.	Leadership	Building local level leadership to make a quick and appropriate decision if and when needed

West Java Provincial Government has a strong commitment to realising the programmes of community protection to create a conducive climate for the creation of security and public orderly in West Java. At the same time, to facilitate the functioning of a democratic society, with political insight and high national integrity, threats, disaster management and community protection to realise political and social stability.

Various problems were encountered related to the protection of the community, especially in disaster management:

- The geographic condition of West Java that is prone to disaster causes the community’s vulnerability to natural hazards and disasters caused by improper environmental management is increasing. This condition has not been matched with effective disaster management either in the pre-disaster stage, during the emergency and post-disaster phase, which includes rehabilitation, relocation and reconstruction.
- Public awareness in anticipating disaster hazard is still low, so it is necessary socialisation and learning or education of disaster management to increase awareness and participation of society in predicting disaster early.
- Community protection (Linmas) activities that have been there in various regions are still not functioning optimally. They face various constraints of limited infrastructure, funds, management and coaching. This condition requires the development of an active and robust community protection management system and encourages and redirects self-security.
- Human resource capability, the community protection unit in disaster management and refugee handling are still not professional. This condition requires the enhancement of the human resources capacity of the community protection unit.
- Inter-agency coordination in disaster management, both horizontally and vertically, is still relatively low. This condition requires the development of system procedures and development of information systems and preparedness in the framework of community protection.

In this context, the empowerment of Linmas personnel is a strategic policy that can be implemented to optimise the role of Linmas institutions in creating a conducive climate of security and social orderly. The empowerment of Linmas should be directed towards improving the competence and professionalism of members of the community, providing infrastructure and facilities support and building a system for maintaining security and public order based on community awareness.

The existence of the Linmas unit has a significant meaning for the process of governance in the region. Therefore, each member of Linmas is expected to have the potential to perform the role, duty and understand to live the function of Linmas. Awareness of the members of the community as one of the implementers of the community protection function that is always ready to cope with various forms of disasters and other disturbances to create a sense of security in the community must still be improved continuously through effective and sustainable Linmas coaching.

Based on the given phenomenon and to realise the efforts of fostering quality Linmas, mainly to see how far the process and the results of the implementation are achieved. It is necessary to conduct research that aims to: (1) establish the mechanism of development of community protection, (2) Identify the support of facilities and infrastructure needed to facilitate the implementation of the tasks and functions of the community, especially in disaster management and (3) formulating a community protection agency that fits regional needs and capacities. With a useful and quality Linmas development policy, it is expected that the West Java Provincial Government can realise the establishment and development of a community protection unit to be more empowered in anticipating and overcoming disasters.

## Research methods

This study used a single case study strategy. There are several different methods, including semi-structured interviews, direct observation, documents, and focus group discussion (FGD). The use of different ways allows triangulation that increases the validity of the study (checking data from various sources, amongst others, through methodological triangulation. The credibility of the data is performed by checking the data to the same source with different techniques (FGD, interviews, then verified by observation and documentation). Data collection uses FGDs from research, including relevant actors from communities and public institutions, to provide a holistic perspective on community protection. This includes residents, city officials, NGO practitioners and members of the community protection unit. In total, 50 people contributed (see Table 2).

**TABLE 2:** Number and types of informants from those involved in focus group discussions.

Informant number	Type of Informant	Number of participants
1	Experts (college)	2
2	Province official (Satpol PP SKPD)	6
3	City or district official (Satpol PP SKPD)	8
4	Head of sub district	8
5	NGO practitioners	6
6	Head of villages	10
7	Linmas (Officers)	10
**-**	**Total (members of FGD)**	**50**

Linmas, community protection; NGO, non-governmental organisation; FGD, focus group discussion; PP SKPD, Pamong Praja police unit.

Data processing follows a strict encoding strategy to find patterns between data. Complete coding manually is applied to identify ‘anything’ and ‘everything’ that is interesting for research purposes.

## Result and discussion

**FIGURE 2:**
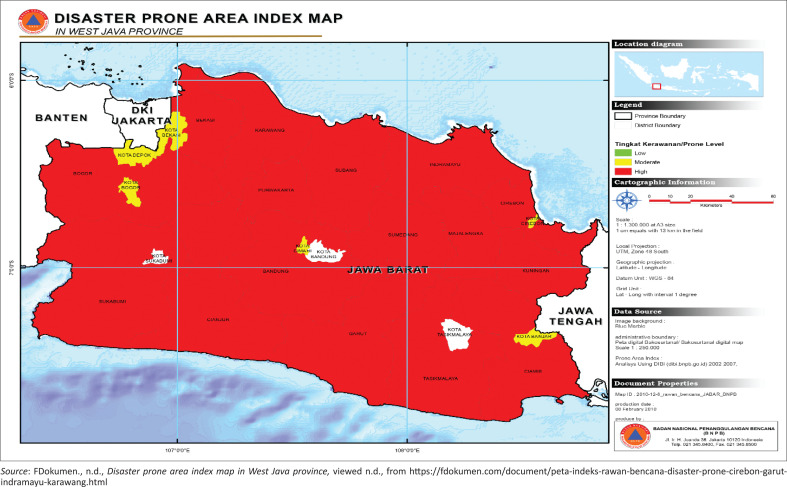
Disaster prone area index map in West Java province.

West Java province is the province with the largest population in Indonesia (18% of the total population in Indonesia) spread across 27 regencies or cities, thus bringing large numbers in the event of a disaster, both fatalities and property. In the earthquake transition, West Java province is on the tectonic earthquake path, which has mountainous topography and river flow. It generally empties into the North Coast region, wherein some areas are prone to flood, landslide, earthquake etc.. West Java is a province with not only a high growth rate but also has a high potential for natural change (see Figure 2).

Based on the data collection of village potentials conducted by the Central Statistics Agency (BPS 2018) related to land disasters in West Java, which occurred in 1824 villages in the last 3 years. More than 15% of the 2014 Podes results. In addition to landslides, which were the most common events, including two other disasters floods and earthquakes that resulted in more than 1000 villages. Floods have occurred in 1185 villages out of 1427 flood-prone villages in West Java in the last 3 years.

Also, West Java province is included in 10 locations of 2015–2019 national priorities in the 2015–2019 National Disaster Management Plan for the National Disaster Management Agency for various types of disasters. The determination of the site of the national priority is the result of an agreement of all Kementrian/Lembaga/Ministry/Institution (K/L) related to the 2013 national meeting. The type of disasters that occur in this region are nationwide priority locations in areas of the earthquake, tsunami, volcanic eruptions, landslides, floods, flash floods, drought, extreme weather, epidemics and epidemics and technological failures. According to the national disaster management agency, Badan Nasional Penanggulangan Bencana (BNPB) (2018), this determination is based on: (1) the number of people and infrastructure exposed; (2) the probability of occurrence for the next five years; and (3) events impact more than two provinces. The potential for massive activities and disasters that occur in Indonesia, especially in West Java, as a buffer for the Capital should be a concern of the government.

Furthermore, BNPB (2018) discussed the West Java region as a disaster supermarket because all kinds of natural hazards have occurred in the area. West Java is an area with a high potential for natural hazards in Indonesia, including floods, tornadoes, landslides, volcanic eruptions, and tsunamis that can occur in the most populous province in Indonesia. There are volcanoes; in the southern region, there is also the potential for tsunamis, environmental damage is very high and in recent years tornadoes often occur. So West Java province is included in the complete category of types of disasters that have occurred. Of course, this has the potential for significant losses as a result. Following is a description of the losses from natural hazards by type in West Java in 2016.

The natural hazards that have occurred have resulted in the loss of both lives and property (Table 3). Consequently, there needs to be a coordinated effort to overcome natural hazards to minimise human casualties. People need protection against disasters, including social, natural and non-natural hazards. It must must be taken seriously, especially for areas that are very prone to accidents as needed as early as possible.

**TABLE 3:** Total losses because of natural hazards by type in West Java, 2016.

Regency or city		Human victim dead	Damaged house				
			Damage		Destroyed	In danger	Drawn
			Light	Severe			
**Regency**							
1.	Bogor	-	837	53	-	-	-
2.	Sukabumi	10	566	306	-	171	-
3.	Cianjur	2	9	13	118	3	11
4.	Bandung	3	5.517	43	-	1	22.399
5.	Garut	34	362	527	359	-	1.370
6.	Tasikmalaya	-	5	57	-	-	-
7.	Ciamis	-	5	14	-	-	-
8.	Kuningan	-	-	-	-	-	-
9.	Cirebon	14	321	89	3	21	3.478
10.	Majalengka	-	-	-	-	-	-
11.	Sumedang	4	771	114	21	43	-
12.	Indramayu	-	-	-	-	-	-
13.	Subang	-	14	4	-	-	-
14.	Purwakarta	-	-	67	-	-	-
15.	Karawang	1	-	3	-	-	-
16.	Bekasi	-	-	-	-	-	-
17.	Bandung Barat	1	41	71	-	4	32
18.	Pangandaran	-	13	32	26	4	32
**City**							
19.	Bogor	2	162	24	-	33	471
20.	Sukabumi	-	7	6	4	-	-
21.	Bandung	-	7	9	5	349	-
22.	Cirebon	-	-	-	-	-	-
23.	Bekasi	-	-	-	-	-	-
24.	Depok	-	-	74	-	-	-
25.	Cimahi	4	38	13	-	-	-
26.	Tasikmalaya	-	-	-	-	-	-
27.	Banjar	-	14	6	-	7	10
**-**	**West Java province**	**75**	**8.689**	**1.525**	**536**	**636**	**27.803**

*Source*: BPS, 2018, *Provinsi Jawa Barat Dalam Angka (Jawa Barat province in figure) 2018*, BPS-Statistics of Jawa Barat Province, Bandung, p. 708.

Regarding institutional capability, the local government is categorised as capable if it has a clear structure, roles, responsibilities and relationships with all other levels of government. Based on the results of the FGDs, the working mechanisms of community protection are described in the following sections.

### Working mechanism of public protection (Linmas)

**FIGURE 3:**
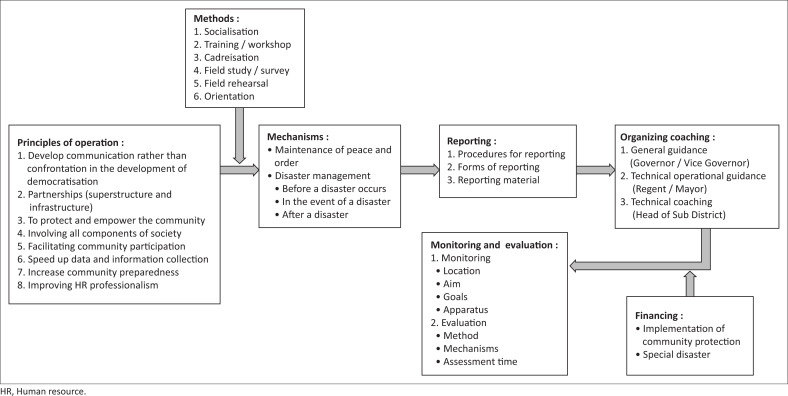
Principle and methods for the implementation of community protection in disaster management.

The working mechanism of community protection, especially related to the implementation of disaster management and refugee handling, is performed in stages starting from the village or sub-district, district, regencyand province. Each level from village to provinces should set up crisis centre as a means of communication and coordination in solving problems precisely, quickly and coordinate following the principles and methods of implementation as illustrated (Figure 3).

The scope of facilitation of provincial government on community protection activity mainly assists in realising professional disaster management:

- Establishment of Community Awareness Systems
  ■ Creating early warning
  ■ Inventories of disaster-prone areas
  ■ Making maps of disaster-prone regions
  ■ Provision of information on disaster-prone areas
  ■ Preparation of permanent procedures for natural hazards management
- Facilitation of the implementation of prevention and rescue against disaster threats, including:
  ■ Facilities for preparing post and disaster preparedness unit
  ■ Formulating a relocation policy for the affected population
- Facilitation of rehabilitation, relocation and reconstruction as a result of disasters, including:
  ■ Provision of relocation assistance for disaster-affected people facilities in settlement arrangement and environment for the new area (relocation)
  ■ Monitoring and evaluation of the implementation.

**FIGURE 4:**
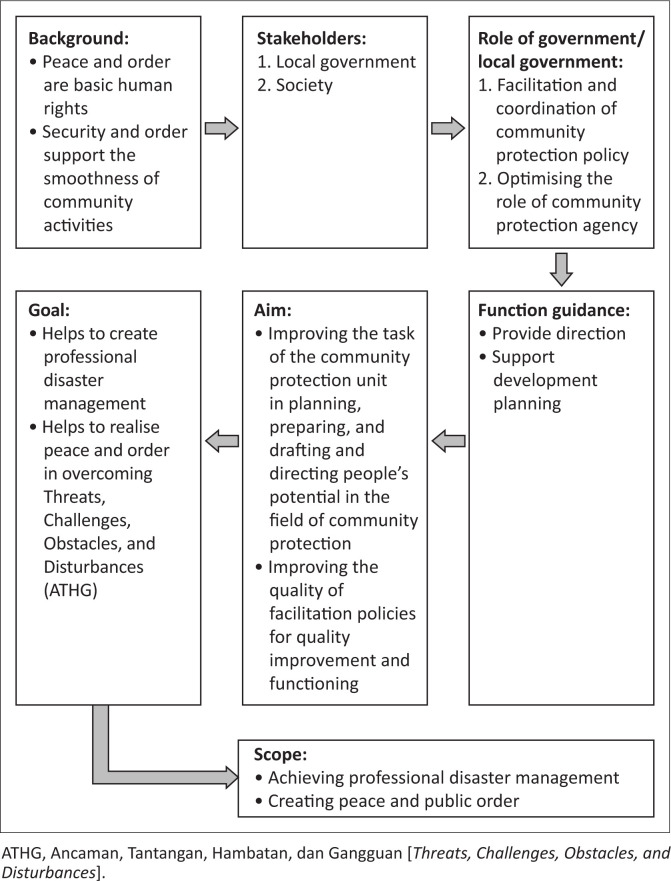
Position and function guidelines for the development of community protection.

The position and functions of guidelines for the development of community protection can be seen (Figure 4).

### Recruitment and empowerment of community protection unit

Communities should be able to play a vital role in making every effort to reduce the risk and impact of disasters. Communities have the power to activate internal resilience capacity to handle and recover areas affected by natural hazards. This research highlights that the city is not just a passive disaster victim. By empowering the community as a person who can help himself and his environment, cooperate, increase confidence, local knowledge and participation as an active agent through a community protection unit.

Table 4 is an illustration of the number of members of community protection in West Java province by Regency City in 2015–2017.

**TABLE 4:** Members Linmas and trained members by regency or city in west Java Province, 2015‒2017.

Regency or city		Linmas members			Trained members			Untrained members		
		2015	2016	2017	2015	2016	2017	2015	2016	2017
**Regency**										
1.	Bogor	4.281	4.340	4.340	2.160	2.160	2.160	2.121	2.180	2.180
2.	Sukabumi	16.961	15.304	15.304	1.101	1.101	1.101	15.860	14.203	14.203
3.	Cianjur	3.600	3.748	3.748	1.436	1.436	1.435	2.164	2.312	2.313
4.	Bandung	5.600	5.600	5.600	3.433	3.433	3.433	2.167	2.167	2.167
5.	Garut	12.262	13.210	13.210	NA	NA	4.250	12.262	NA	8.960
6.	Tasikmalaya	8.499	8.817	8817	2.624	351	351	5.875	8.148	8.466
7.	Ciamis	8.856	5.474	5474	3.030	3.030	3.030	5.826	5.826	2.444
8.	Kuningan	3.285	3.380	3.380	1.114	376	376	2.171	2.671	3.004
9.	Cirebon	4.982	4.539	4.539	851	851	851	4.131	4.131	3.688
10.	Majalengka	3.367	3.430	3.430	1.035	1.035	1.035	2.332	2.332	2.395
11.	Sumedang	2.830	5.106	5.106	824	824	824	2.006	2.106	4.282
12.	Indramayu	1.560	1.585	1.585	1.560	1.529	1.529	NA	NA	56
13.	Subang	3.626	3.530	3.530	1.707	1.707	1.707	1.919	1.919	1.823
14.	Purwakarta	2.880	2.880	2.880	387	387	589	2.493	2.493	2.493
15.	Karawang	3.079	3.090	3.090	NA	NA	1.212	3.079	NA	1.878
16.	Bekasi	1.870	1.870	1.870	1.870	1.870	1.870	NA	NA	NA
17.	Bandung Barat	3.795	3.795	3.795	1.300	1.300	1.300	2.495	2.485	2.495
18.	Pangandaran	1.856	1.886	1.886	NA	NA	NA	1.856	NA	NA
**City**										
19.	Bogor	3.709	3.739	3.739	NA	NA	NA	NA	NA	NA
20.	Sukabumi	2075	2.075	2.075	1.471	1.471	1.471	604	441	604
21.	Bandung	11.577	11.782	11.782	11.577	NA	11.577	NA	NA	205
22.	Cirebon	1.328	1.100	1.100	412	412	412	916	845	688
23.	Bekasi	1.677	1.736	1.736	244	244	244	1.433	1.433	1.492
24.	Depok	634	630	630	242	242	242	392	407	388
25.	Cimahi	2.105	1.885	1.885	143	143	143	1.962	1.571	1.742
26.	Tasikmalaya	3.690	3.864	3.864	179	179	179	3.511	2.534	3.685
27.	Banjar	944	1.029	1.029	244	244	244	700	703	785
-	Total	120.928	119.424	119.424	NA	NA	NA	NA	NA	NA

LINMAS, community protection; NA, not available.

In Table 4 it can be seen that the number of community protection members in each region varies greatly and that number is still inadequate if related to the needs of members of Linmas in each village, especially members of Linmas who have been trained. There are still many members of ‘Linmas’ who have been recruited and yet have not received training. Training is part of the empowerment of members of Linmas, which includes: increasing the ability of Linmas in disaster management, increasing the capacity of Linmas to prevent social vulnerability and early awareness of situations that disturb public order and peace and organising regional elections.

As already explained, the members of ‘Linmas’ are domiciled as supporting elements of the Provincial Government and Regency or City Government in carrying out disaster management activities to reduce and minimise the consequences of disasters and to participate in maintaining security, peace and order of the community and human society. The recruitment of *Satlinmas* members in Villages (rural or urban) is carried out by the village head. Enlistment is voluntary and open to all citizens.

The organising of community protection units (*Satlinmas*) in West Java was conducted by recruiting citizens to become members of a *Satlinmas* in villages by the village head voluntarily and openly to all citizens. The recruitment of *Satlinmas* members is made to the people who meet the following requirements:

- indonesian citizens
- the deity to God Almighty
- true to Pancasila and the 1945 Constitution of the State of the Republic of Indonesia
- aged at least 18 years and married
- minimum junior secondary education level and equivalent
- physically and mentally healthy
- residing in the local village.

Citizens who fulfil the requirements are designated as public protection units with the decision of the Regent or Mayor signed by the District or Municipal Pamong Praja Police Unit Head. The term of the *Satlinmas* membership ends up to the age of 60 years or is dismissed for the following reasons:

- death
- resigned at his or her request
- moved to another domicile
- no longer met health requirements
- committed a disgraceful act
- committed a criminal offense that has obtained permanent legal force.

*Satlinmas* is an auxiliary element of the provincial and district or municipal governments in implementing disaster management activities to reduce and minimise the impact of disasters and to maintain security, peace and order and social events (see Figure 3). Head of the community protection unit, ex officio held by the Village Head or Lurah, who then appointed the head of the task force. In stages, the administrator of the task force selects a squad team of at least 10 people. There are five squad commanders, amongst others:

- preparedness and early alert teams
- security team
- first aid teams on victims and fires
- rescue and evacuation teams
- public kitchen team.

Based on the results of interviews and FGDs, data and information were obtained to perform the main tasks and functions established units implementing features by the respective handling areas. The services of each sub-district Task Force can be seen (Table 5).

**TABLE 5:** Duties of the community protection unit at the sub-district level.

Number	The sub-fields and tasks handled in each field
1.	Implementing task force on early prevention covers activities: identify disaster-prone areas throughout the work area of the kecamatan creating disaster-prone maps throughout the sub-district work area analysing disaster-prone areas throughout the work area of the subdistrict increasing community awareness through early warning efforts against possible disaster and disruption across all work areas of the subdistrict counseling and dissemination of disaster-prone areas and disaster characteristics that will occur through various print and electronic media establishing an alternative location for the evacuation of victims and disruption creating posts in disaster-prone areas to give warning coordinating with other implementing units at district and provincial levels assisting other unit tasks in the event of a disaster and disturbance.
2.	The rescue unit includes activities: preparing the potential of community and community units to address disasters, disruption and handling of refugees coordinating communities in the preparation of disaster management tools and facilities, disruption and handling of refugees searching for and rescue victims from disasters providing first aid to victims as a result of disasters and disruptions displacing disaster victims and harassment placing disaster victims in temporary shelters in a safe location.
3.	Unit of health, covering activities: efforts to enhance health, prevent and eradicate disease or outbreaks and to rehabilitate the media throughout the working areas of the subdistrict preparing paramedical data and health facilities or FSP providing first aid or emergency of disaster victims and disturbances referring to disaster victims who suffered physical, psychological and social disorder to the hospital or related rehabilitation institution.
4.	The public works unit covers activities: preparing temporary shelters for disaster victims and harassment performing a light reconstruction of social facilities and public facilities affected by the disaster securing the disaster area investigate the number of disaster victims and estimate the amount of loss redirecting disaster victims to their original location, community settlements or transfer of safe areas rehabilitate the affected areas.
5.	Social unit, covering activities: conducting emergency welfare efforts – opening up a communal kitchen, emergency shelter, emergency clothing receiving, managing and distributing and accountable for assistance identifying potential sources of assistance providing motivation and counseling to the affected communities.

FSP, free servise program.

On the other hand, the empowerment of community protection personnel is a strategic policy that can be carried out to optimise the role of community protection institutions in creating a conducive climate of security and social orderly. The empowerment of community protection (Linmas) is directed towards improving the competence and professionalism of members of the community protection, providing infrastructure and facilities support and building a disaster management system based on community awareness.

On the other hand, the recruitment and empowerment of members of ‘Linmas’ in handling disasters is essential and must be prioritised given that most regions in Indonesia, including West Java, are disaster-prone areas. Linmas members can play an important role before, during and after disaster because the ‘Linmas’ member knows the community very well. If this condition is fulfilled, it will undoubtedly be strong support for the regional government, given that two critical areas are underexplored in terms of the role of local government in managing disasters. Firstly, the issue has been examined mostly in the context of local government in developed countries and insufficient attention has been paid to the local government in developing countries. Secondly, the resource capabilities of local government in managing disasters in every stage (pre-, during and post-disaster events) have not been examined. Indeed, in recent years many local government bodies have faced difficulties in dealing with disasters because they have inadequate knowledge and capabilities to manage hazards. This is in line with what Kusumasari et al. (2010) pointed out that the competitive factors of human resource-related skills are evident when local governments have sufficient personnel, appropriate tasks, delegations and division of labour within the organisation to manage the disaster. The critical success factors that contribute to policies for capabilities related to effective implementation are the availability of appropriate policies, rules and regulations to make decisions to mobilise resources and engage relevant public and private organisations.

## Conclusion

The mechanism for the development of community protection shall be implemented in an integrated and hierarchical from the village, sub-district, district or city, provincial, even to the national level. Support facilities and infrastructure to facilitate the implementation of duties and functions of the community, especially in disaster management allocated and budgeted not only at the district or city level but also the provincial level. Active community protection institutions should be by local needs and capacities.

### Recommendation

The community protection organisations need to be managed by individual units in which they have minimum skills and skills in the field:

- early prevention
- peace, order and safety
- health and psychologists
- public and social work.

Ability and skills in each area of community protection are expected also attached to each agency that has authority in their respective regions. The community protection organisation is directed as a center for crisis management in the community.

The community protection organisation is always on standby in performing its duties, both in a stable and an emergency so that the actions taken during stable conditions are: to conduct a simulation of each problem handling and capacity building, skill continuously. In the event of the emergency condition of a fast, proper and coordinated organization, treat the emergency condition.

The practical implication of this research is that the local government should empower the community protection unit through the regional work unit or related stakeholders in conducting training and facilitation of training and improving skills so that they can carry out their duties better.

This research covers the protection community only in West Java province, and the findings cannot be generalised to others. Even though the study provides some contributions to proposed a mechanism of evolution of community protection by the local government and can be a barometer for other regions, the Future research needs to be examined in a more full administrative area by involving stakeholders in disaster management that is more comprehensive and integrative, including the involvement of BNPB or the regional disaster management agency, Badan Penanggulangan Bencana Daerah (BPBD), as an institution that has full authority in disaster management.
